# Gender and Age Patterns in NSGA Swim Competitions

**DOI:** 10.1155/2022/8459520

**Published:** 2022-07-18

**Authors:** David P. Doane, Lori E. Seward, Kevin Murphy

**Affiliations:** ^1^Department of Decision and Information Sciences, Oakland University, Rochester, MI 49309, USA; ^2^Leeds School of Business, University of Colorado, Boulder, CO 80309, USA; ^3^Department of Economics, Oakland University, Rochester, MI 49309, USA

## Abstract

We estimate two common nonlinear models (quadratic and semilog) and one new model (exponential) of the time-age relationship in 500-yard freestyle swim times in the U.S. National Senior Games (ages 50 and up) in six biennial NSGA competitions (2009, 2011, 2013, 2015, 2017, and 2019) for 468 men and 587 women. We use OLS and quantile regression (25%, 50%, and 75%) separately for each gender. The semilog model predicts faster times than the quadratic or exponential models. Our hypothesis that women slow down faster than men after age 50 is supported by both models as well as by our unique within-gender comparisons. Our findings of a nonlinear performance decline agree with studies of elite swimmers (Olympic, FINA). Our first-time study of NSGA data provides new guidelines to inform senior competitors. Our findings will assist trainers and community organizations that support NSGA competitions to promote a healthy senior lifestyle.

## 1. Introduction

Elite swimmers (Olympic, FINA, U.S. Masters) have been studied extensively to establish performance limits and to reveal patterns by age and gender. Most studies cover a wide range of ages [1–8], although some focus on older swimmers [9–13]. Specific attention has been given to how records have improved over time [4, 14–19]. Longitudinal studies [6, 9] are less common than cross-sectional or hybrid studies (see [20] for a good summary). Studies emphasizing the physiology of aging in sports [21], including nonswimming events [10, 22, 23], raise methodological issues that will be discussed in this paper.

Our research seeks to inform and encourage individuals, trainers, and organizations that support senior swimming and healthy aging. For most recreational senior swimmers, ultimate physiological frontiers and Olympic records are an unapproachable asymptote that offers no realistic target to assess their own progress. To provide performance benchmarks that inform and guide a broader cross-section of senior competitors, we examined published times in an endurance swim event (500-yard freestyle short course) in six consecutive competitions of the National Senior Games Association (NSGA).

How does NSGA data differ from elite competition data? First, only those aged 50 and over can compete. Second, while some NSGA athletes may be elite, many are novices who are attracted to local NSGA competitions through senior centers and community organizations that focus on healthy aging and senior physical and mental health. For example, the sponsors of Michigan Senior Olympics NSGA include a local university, county parks and recreation, health care providers, and senior living groups [24]. NSGA-sponsored games and self-organized teams provide a social nexus for older adults with community involvement and volunteer opportunities (e.g., setup, judging, coaching) for nonseniors and seniors alike, publicized by local media [25].

To participate in NSGA nationals, one must qualify in an NSGA-approved state competition and be at least 50 years old in the qualifying year. Medal sports include archery, badminton, basketball, bowling, cycling, golf, horseshoes, pickleball, race walk, racquetball, road race, shuffleboard, softball, swimming, table tennis, tennis, track and field, triathlon, and volleyball. In most sports, including swimming, the top 4 finishers in each age group (50–54, 55–59,…) in state competitions are eligible to compete in the nationals.

We hypothesize (a) a nonlinear relationship between age and performance and (b) a greater slowdown for women than for men after age 50. We investigate these hypotheses using both conventional and novel models, methods of estimation, and comparisons of performance as a function of gender and age.

## 2. Methods

Table 1 shows the number of entrants who completed the 500-yard freestyle event in six consecutive NSGA competitions. Although as many as 200 could qualify within each gender (50 states times 4 per state), the actual participant counts are lower. Participation in the national games is affected by the host city's attractiveness and travel costs. The NSGA summer games last two weeks, so hotel and meal costs may discourage athletes who feel that their medal chances are too low to justify a trip. Moreover, athletes can qualify in up to six events (including track and field and other sports), so swimmers may choose only events where they have the best chance for a medal. Senior athletes may also face health issues that force them to drop out.

Table 2 shows the age distribution of participants in our data set. As expected, there are fewer participants in higher age categories (only one woman and no men in the 95–99 age category). The tabulation of individual participant names (with correction for inconsistent name spelling and cross-checking of ages) revealed that 215 swimmers had competed in multiple years. We assigned each swimmer a unique ID, yielding 678 unique participants. Our data, therefore, has a longitudinal as well as a cross-sectional component.

## 3. Results

**Figure 1:**
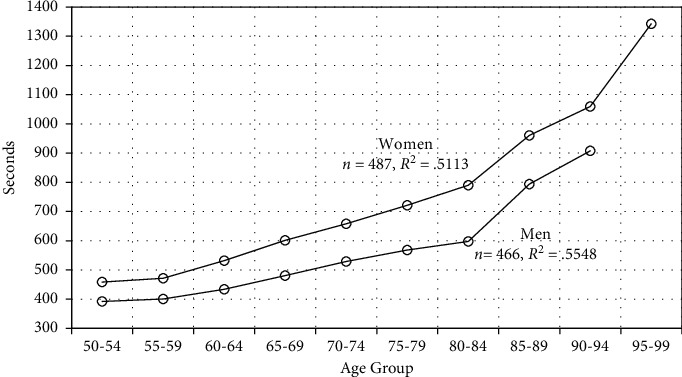
Predicted swim times by age group.

We estimate the relationship between swim time (Time) and age (Age) for each gender separately (as opposed, say, to regression with a gender binary and interaction). All six years are pooled because our exploratory regressions revealed no consistent time trends. For men, exploratory regression revealed two extremely slow times (standardized residuals of 7.05 and 7.76), which we chose to omit in subsequent regressions. For women, one extremely slow swimmer (standardized residual 5.27) was retained as its effect on the overall fit was modest.  Figure 1 shows a model-free regression with *time* as the response and predictor dummy (0–1) variables for each 5-year age group. Average times increase more than linearly with age, with a possible trend break starting in the 80–84 age group.

Table 3 shows estimates of three continuous models of the time-age relationship. We used ordinary least squares (OLS) regression for the quadratic and semilog models and Excel's Solver for the exponential model. For comparability, we calculate *R*^2^ and standard error from fitted model predictions for semilog models (semilog residuals are reported in log form) and Solver models (gradient estimation produces no residuals).

The quadratic form implies a turning point. Equating *d*(Time)/*d*(Age) to zero and solving we get Age = 39 for the women and Age = 51 for the men as the “best” age. However, because our sample did not cover swimmers below age 50, the utility of this result is doubtful. Yet this finding is consistent with research showing that elite women swimmers start to slow down at an earlier age than male swimmers [13, 23]. Regarding gender, the semilog slope for women (0.0209 Age) exceeds that of men (0.0196 Age), implying a more rapid increase in time with increasing age.

An attraction of the Solver (exponential form) is that it allows the Age exponent to vary. We set its origin at 50 to specify a monotonic increase in swim times after age 50. Our Solver exponents are less than 2, in contrast to the quadratic model whose quadratic term (Age^2^) always has an exponent of 2. The exponent for women (Age^1.699^) exceeds that of men (Age^1.639^), which suggests that women's times increase more rapidly than men's after age 50.

Table 4 shows that semilog predicted times are consistently less than those from the quadratic, especially after age 80. For women, OLS and Solver give similar predictions. Despite their differences in form, Solver's predictions closely match the quadratic at all ages for women and for men, except at the highest and lowest ages. As far as we know, no previous research has explored allowing the age exponent to vary in this way. This topic may merit further scrutiny. However, because the quadratic is more commonly used and is easier to estimate, we will omit the Solver (exponential) model from further discussion here.

Next, we used quantile regression to estimate 25%, 50%, and 75% quantiles (Table 5). An advantage of quantile regression is that it lessens the impact of unusual observations (e.g., extremely slow swimmers) as well as allowing estimation ofspecific percentiles. Because of sparse data for higher age groups, a finer breakdown (e.g., deciles) is not practical. Because quantile regression minimizes the sum of the absolute residuals rather than the sum of the squared residuals, the software does not report a comparable standard error or *R*^2^. If needed, those statistics can be generated by making predictions and summing the squared errors.

For both genders, the semilog slope (coefficient of the Age term) of the 75th percentile (the slowest swimmers) exceeds that of the 25th percentile (fastest swimmers). That is, the fastest swimmers' performance declines more slowly with Age. As with the OLS estimates, the coefficients of the age terms in the semilog model suggest that women's times increase faster than men's after age 50.

**Figure 2:**
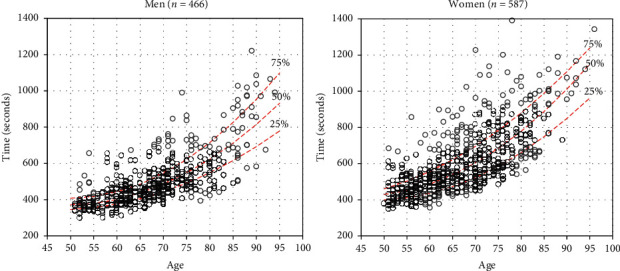
Quantiles for fitted quadratic model.

Figure 2 shows quantiles for the quadratic plotted for each gender. An aspiring swimmer who hopes to compete in the NSGA Summer Games could use these quantiles to assess his/her relative standing against recent competitors in national NSGA competitions. Given the typical number of NSGA competitors in each age group, a swimmer in the fastest 25 percent would have a reasonable chance of placing in the top 8, thereby qualifying for recognition on the winner's dais (although only the top 3 receive medals).

Table 6 illustrates that the OLS prediction (conditional mean) generally lies above the 50% quantile prediction (conditional median). The 50% coefficient estimates are less affected by high extremes (unusually slow swimmers).

Table 7 shows that predictions from the semilog quantiles match those of the quadratic quantiles up to about age 75 but diverge noticeably for higher ages (especially for men).

**Figure 3:**
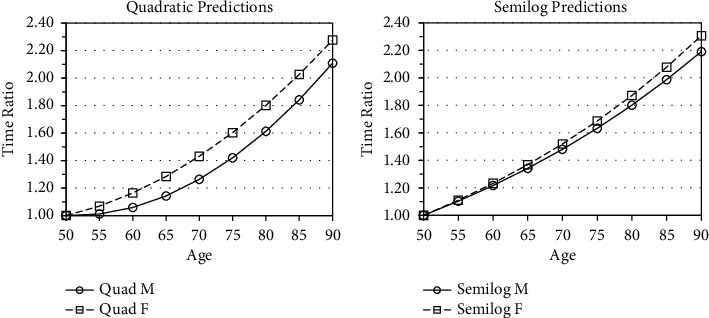
Predicted ratios (age 50 = 1.00).

We hypothesize that female swimmers slow down at a greater rate than male swimmers after a certain age. To focus explicitly on age-related change within each gender (as opposed to comparisons *between* genders), we express predicted times relative to the age of 50 using an index (age 50 = 1.00). The quadratic model (Figure 3(a)) does show a more rapid slowdown for women, at least until age 70. The semilog model (Figure 3(b)) agrees that women slow down more rapidly with age, on average. Thus, the evidence supports our hypothesis.

## 4. Discussion

To capture nonlinearity, other researchers have often used the quadratic model [2, 3, 9–11]. Other methods include linear regression [1, 4], the semi-log model [6], hybrid models [4], and GLM [22]. In most studies (including ours), data are sparse at the highest ages, a problem noted by Rubin et al. [6]. Studies of the 800m or 1500m events [3, 8] offer an approache somewhat comparable to ours (i.e., endurance events), although for elite athletes.

Other studies, e.g., [22], agree with our conclusion that women's performance declines faster than men's after age 50. Several researchers have suggested a possible point of inflection in athletic performance near age 70 or a bit later [2, 3, 5, 8, 9, 23, 27–29], as we have also suggested. A point of inflection implies a continuous monotonic function relating swim time to age. Mathematical issues aside, what physiological breakdown might create a discontinuity? Lazarus and Harridge [28] ask *why* there should be “an increased rate of decline occurring in many events at 70 and 80 years of age” (Leibniz's *natura non facit saltus*). They consider various explanations, concluding it is likely that “this breakpoint is a reflection of a decline in the synchrony and integration of the systems that go to make up whole-body performance” of physiological mechanisms [19, 31–33]. Yet, the search for a single predictive model may be moot given the wide variation in competitive swimming performance at later ages. For swimmers aged 50–75, any of the several continuous models described in our paper would provide useful benchmarks.

While the role of gender in swimming has been studied [2, 8, 13, 18, 26], along with the “peak” age question [2, 26, 27], we feel that our *within-gender* comparison provides a clearer focus on the role of gender on age-related performance decline.

## 5. Conclusion

Our use of quantile regressions and the semi-log model adds new perspectives on model estimation. Our analysis complements extant research on elite athletes showing that swim times are best predicted using a quadratic model based on age and gender, with more rapid slowing after age 50, especially for women. Our *within-gender* performance comparisons offer a new way to visualize performance declines by gender. Our study of NSGA competitors adds a new dimension to existing studies of FINA, Masters, and Olympic athletes, which we believe will inform and encourage a broader spectrum of senior swimmers. Over time, NSGA swim performance may become more comparable with Master's events as better-trained entrants enter the games [34]. We know of one other study [30] using NSGA data, although it was for track and field events. We hope that our work will stimulate the analysis of other NSGA sports.

## Figures and Tables

**Table 1:** NSGA biennial venues and 500-yard freestyle competitors.

2009	San Francisco, CA	*n* = 199 (89 men, 110 women)
2011	Houston, TX	*n* = 189 (76 men, 113 women)
2013	Cleveland, OH	*n* = 178 (83 men, 95 women)
2015	Minneapolis, MN	*n* = 158 (78 men, 80 women)
2017	Birmingham, AL	*n* = 154 (69 men, 85 women)
2019	Albuquerque, NM	*n* = 177 (73 men, 104 women)

**Table 2:** Swimmers by age group.

Age	Men	Women	Total
50–54	34	53	87
55–59	54	88	142
60–64	89	106	195
65–69	91	93	184
70–74	81	103	184
75–79	53	71	124
80–84	32	52	84
85–89	26	13	39
90–94	8	7	15
95–99	--	1	1
Total	468	587	1055

**Table 3:** OLS regressions and solver estimates.

	Fitted model	*R * ^2^	Std err
Men (*n* = 466)			
Quadratic	Time = 1142.9–29.338 Age + 0.28783 Age^2^	0.5556	97.58
Semilog	Time = exp (4.8573 + 0.019601 Age)	0.5305	100.19
			
Solver	Time = 363.582 + (Age−49)^1.6394^	0.5469	98.53
Women (*n* = 587)			
Quadratic	Time = 764.03–17.894 Age + 0.22788 Age^2^	0.5169	126.51
Semilog	Time = exp (4.9730 + 0.020885 Age)	0.5099	127.33
Solver	Time = 450.301 + (Age−49)^1.69903^	0.5163	126.59

*Note.* Quadratic and semilog results are from Stata's *reg* procedure with bootstrap standard error. All regression coefficients are highly significant. Solver coefficient estimates are from Excel's Generalized Reduced Gradient method.

**Table 4:** Predictions from three models.

	Men (*n* = 466)				Women (*n* = 487)		
Age	Quad	Semilog	Solver		Quad	Semilog	Solver
50	396	343	365		439	410	451
60	419	417	415		511	506	509
70	500	507	511		628	623	627
80	638	617	642		791	768	792
90	834	751	804		999	946	1000

**Table 5:** Quantile regression benchmarks.

Men (*n* = 466)		
Quantile	Quadratic model	Semilog model

25%	Time = 769.30–17.994 Age + 0.19044 *Age*^2^	Time = exp (4.9078 + 0.017181 Age)
50%	Time = 1037.10–26.872 Age + 0.27113 Age^2^	Time = exp (4.8508 + 0.019357 *Age*)
75%	Time = 1259.77–34.071 Age + 0.34075 Age^2^	Time = exp (4.7608 + 0.022424 *Age*)
Women (*n* = 587)		
Quantile	Quadratic model	Semilog model
25%	Time = 975.222–24.163 Age + 0.25293 Age^2^	Time = exp (5.0279 + 0.017915 Age)
50%	Time = 1006.75–26.088 Age + 0.29046 Age^2^	Time = exp (4.8820 + 0.021932 Age)
75%	Time = 639.46–14.587 Age + 0.21992 Age^2^	Time = exp (4.9627 + 0.022685 Age)

*Note.* Quantiles are estimated using Stata's *bsqreg* procedure with bootstrapped standard errors.

**Table 6:** Predicted times for selected ages using quadratic model.

Age	Men					Women			
	OLS	25%	50%	75%		OLS	25%	50%	75%
50	396	346	371	408		439	399	429	460
60	419	375	401	442		511	436	487	556
70	500	443	485	544		628	523	604	696
80	638	549	623	715		791	661	779	880
90	834	692	815	953		999	849	1012	1108

**Table 7:** Predicted times for selected ages using semilog model.

Age	Men					Women			
	OLS	25%	50%	75%		OLS	25%	50%	75%
50	343	320	337	359		410	374	395	445
60	417	379	408	449		506	447	492	558
70	507	451	496	561		623	535	612	700
80	617	535	601	703		768	640	762	878
90	751	635	730	879		946	765	949	1101

## Data Availability

NSGA biennial competition swim times are publicly accessible (https://nsga.com/nsgresults/) by year, event, gender, and age group. Formatted data (Excel, CSV, or PDF) from this study are available upon request from the author David P. Doane.
